# Cue-Reactors: Individual Differences in Cue-Induced Craving after Food or Smoking Abstinence

**DOI:** 10.1371/journal.pone.0015475

**Published:** 2010-11-10

**Authors:** Stephen V. Mahler, Harriet de Wit

**Affiliations:** 1 Department of Neurosciences, Medical University of South Carolina, Charleston, South Carolina, United States of America; 2 Department of Psychiatry and Behavioral Neuroscience, University of Chicago, Chicago, Illinois, United States of America; University of Chicago, United States of America

## Abstract

**Background:**

Pavlovian conditioning plays a critical role in both drug addiction and binge eating. Recent animal research suggests that certain individuals are highly sensitive to conditioned cues, whether they signal food or drugs. Are certain humans also more reactive to both food and drug cues?

**Methods:**

We examined cue-induced craving for both cigarettes and food, in the same individuals (n = 15 adult smokers). Subjects viewed smoking-related or food-related images after abstaining from either smoking or eating.

**Results:**

Certain individuals reported strong cue-induced craving after both smoking and food cues. That is, subjects who reported strong cue-induced craving for cigarettes also rated stronger cue-induced food craving.

**Conclusions:**

In humans, like in nonhumans, there may be a “cue-reactive” phenotype, consisting of individuals who are highly sensitive to conditioned stimuli. This finding extends recent reports from nonhuman studies. Further understanding this subgroup of smokers may allow clinicians to individually tailor therapies for smoking cessation.

## Introduction

Drug and food rewards are believed to act upon similar neural circuits, and mounting evidence supports the idea that food, as well as drugs can be addictive. In addition to their primary reinforcing properties, drugs and food also establish strong Pavlovian associations with the stimuli, or cues, that predict them [1], [2], [3], [4], [5], [6]. In humans, these cues elicit strong subjective craving states, especially when people are in a food or drug deprived state. In nonhumans, the cues elicit robust drug or food seeking in animals trained to make appropriate responses. A growing body of evidence indicates that cue-induced seeking of food or drugs share overlapping neurochemical and neuroanatomical substrates (see [7] for a review).

Interestingly, preclinical evidence suggests that there are pronounced and reliable individual differences in the propensity to approach cues that predict either food or cocaine [8], [9], [10]. Certain animals are particularly susceptible to the attractive and salient qualities of food-predictive cues, and these same animals show stronger appetitive responses to drug cues [11]. Thus, animals vary in the degree to which Pavlovian cues come to exert control over reward-appetitive behavior, regardless of the type of reward. This subpopulation of individuals with strongly cue-driven behavior may also exist in humans. That is, humans with ‘cue-reactive’ phenotypes may be at increased risk of developing addictive disorders, or be at increased risk of relapse to drug or binge eating disorders following treatment [12], [13].

Here we examined whether cue-induced craving for food was correlated with cue-induced craving for cigarettes in humans. We hypothesized that certain individuals would be more reactive to both food-related and smoking-related cues, suggesting that there is a human phenotype corresponding to sensitivity to Pavlovian reward-associated stimuli.

## Materials and Methods

These data were collected in the context of another study [14], approved by the University of Chicago Institutional Review Board, and all subjects provided written informed consent to participate in this study. Healthy male and female smokers [n = 15 (6 female); age: m(SD) = 25(7) years; 10 Caucasian, 3 African-American, 2 Asian-American; cigarettes/day: m(SD) = 18(5)] participated in a four session study, in which they A) abstained from smoking for 18 hrs, B) abstained from eating for 18 hrs, C) abstained from eating and smoking, or D) freely smoked and ate prior to and during sessions. Sessions were held in randomized order, and separated by at least one week. On each session, subjects were allowed to acclimate to the lab for 2 hrs, then food and smoking abstinence was verified with breath CO and urine ketone tests, and subjects were asked to rate baseline food and smoking cravings. They then viewed blocks of food-related and drug-related images (individually tailored for each subject on an initial orientation session, to maximize craving induction during test sessions), and rated their cravings for food and cigarettes. For details of experimental procedures and verification of the efficacy of cue-induced craving and abstinence procedures see [14].

We examined cue-induced craving for nicotine and food by measuring the change in craving from before to after the cue presentations. The cues consisted of 112 sec blocks of smoking, food, and neutral cues (28 of each, 4 sec apiece), and each block was followed by a period in which subjects reported their food and smoking cravings using versions of the well validated questionnaires the “Questionnaire of Smoking Urges (Brief)” [15], and the “Pittsburgh Appetite Test” [16], [17]. We examined Pearson correlations between smoking and food craving elicited by cues following smoking and food abstinence. We also examined smoking and food craving reported prior to cue presentations in the four abstinence conditions.

## Results

**Figure 1:**
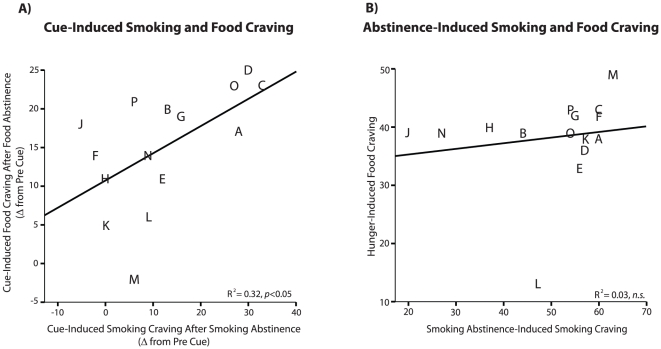
Correlations between craving for cigarettes and craving for food. Cue-induced cravings for cigarettes and food were correlated, but abstinence-induced cravings were not. A.) Food cravings elicited by food cues were correlated with smoking cravings elicited by smoking cues, when subjects were hungry and smoking-abstinent, respectively. B.) In contrast, cravings induced by food abstinence or smoking abstinence alone (without cue presentations) were not correlated.

Participants who reported greater cue plus abstinence-induced food craving also showed higher cue plus abstinence-induced smoking craving (r = .57, *p*<0.05; Figure 1a). In contrast, baseline (pre-cue) levels of craving for food and cigarettes were not correlated when subjects were either non-deprived (r = 0.28, *n.s.*), or deprived of both food and cigarettes (r = 0.29, *n.s.*). Although food and smoking abstinence predictably increased craving for food and cigarettes, respectively, this abstinence-induced craving for food and cigarettes before presentation of the cues was not correlated (r = 0.16, *n.s.*, Figure 1b). Similarly, smoking and food cues only elicited low levels of craving when participants could freely smoke and eat during the sessions, and these cravings were not correlated (r = −0.08, *n.s.*). These findings suggest that human smokers vary specifically in their sensitivity to cue-induced craving when abstinent, suggesting that there may be a “cue-reactive” subgroup of smokers that are particularly susceptible to reward craving elicited by conditioned stimuli, but not by hunger or smoking withdrawal alone.

## Discussion

These findings support the idea that certain individuals are especially susceptible to drug and food-associated Pavlovian stimuli. The same subset of smokers that showed strong subjective craving responses to food cues when hungry also showed strong cue-induced smoking craving when deprived of cigarettes. This effect is not likely to be a product of introspective ability or reporting bias, since food and smoking cravings were not correlated in the absence of cues.

The findings parallel recent preclinical findings. Rats vary in their propensity to exhibit appetitive behaviors directed at Pavlovian food-predictive cues, and the same rats that attribute the most incentive salience upon food-predictive cues also respond most to drug cues [11]. Moreover, hunger specifically potentiates cue-triggered food seeking in these cue-reactive rats [18], similar to our observation here that abstinence from food or smoking potentiates cue-triggered craving for these substances particularly strongly in cue-reactive subjects. There is evidence that this propensity is genetically controlled, mediated in part by dopamine, and strongly predicts drug-induced behavioral markers thought to model human addiction [8], [19], [20]. Here, we show that smokers who reported strong cue-induced smoking craving also tend to report strong food cue-induced craving when in appropriate deprivation states, suggesting that there may be a generally “cue-reactive” phenotype in humans, as well as in nonhumans.

Future research will be required to determine the nature and generality of these variations in cue-reactivity. It remains to be determined whether these variations extend to cue-induced craving for other drugs and other natural rewards, whether cue-reactive drug users are more likely to relapse to drug use after quitting, or whether they respond differently to treatment strategies. In addition, it would be useful to explicitly pair smoking and food with otherwise neutral stimuli in the lab, and examine conditioned responses to these cues, to replicate these results in a paradigm more directly analogous to preclinical studies, and to examine genetic or other factors that might further characterize the phenotype of these “cue-reactive” smokers.

Identification of a subgroup of smokers who are particularly reactive to cues for both drug and natural rewards would help in understanding individual differences in smoking motivations, and for planning individualized treatment interventions. Most notably, the close parallel between these findings and preclinical evidence that animals differ in reactivity to cues suggests that this is a rewarding avenue to pursue translational studies on the role of individual differences in cue reactivity in addiction.
